# Sea cucumbers of the Arabian Peninsula and Iran – A review of historical and current research trends

**DOI:** 10.1016/j.sjbs.2021.10.001

**Published:** 2021-10-06

**Authors:** Amani Al-Yaqout, Manickam Nithyanandan, Faiza Al-Yamani, Mohammad Al-Kandari, Musaad Al-Roumi, Ali Al-Baz

**Affiliations:** Environment and Life Sciences Research Center, Kuwait Institute for Scientific Research, P.O. Box 1638, Salmiya 22017, Kuwait

**Keywords:** Holothurians, Arabian Gulf, Diversity, Fisheries, Aquaculture, Ecotoxicology, Bioprospecting

## Abstract

Sea cucumbers are benthic marine invertebrates with immense ecological and commercial value. Processed sea cucumbers known as “*Beche-de-mer*” are a delicacy in southeast Asian countries with an ever-increasing demand depleting wild stocks on a global scale. Aquaculture techniques are well developed for commercially important species (e.g. *Holothuria scabra*) to aid in conservation and trade. In the Arabian Peninsula and Iran, where the major land mass is surrounded by marginal seas (Arabian Gulf, Gulf of Oman, Arabian Sea, Gulf of Aden, and Red Sea), studies on sea cucumbers are rather limited and its economic value is underestimated. Historical and current research trends indicate impoverished diversity (82 species) due to environmental extremes. Artisanal fisheries exist for the sea cucumbers of Iran, Oman, and Saudi Arabia, with Yemen and United Arab Emirates (UAE) playing a key role in collection and export to Asian countries. Stock assessment and data on export indicates depletion of natural stocks in Saudi Arabia and Oman. Aquaculture trials of high value species (*H*. *scabra*) were successful in Saudi Arabia, Oman and Iran with prospects for further expansion. Research on ecotoxicological properties and bioactive substances conducted in Iran demonstrates an immense research potential. Molecular phylogeny, biology, use in bioremediation, and characterisation of bioactive compounds were identified as potential gaps in research. Expanding aquaculture operations could revive exports and recuperate damaged stocks through sea ranching. Furthermore, regional cooperation, networking, training, and capacity building could help fill the gaps in sea cucumber research, which will aid in its effective conservation and management.

## 1 Introduction

Sea cucumbers, or Holothurians, are marine invertebrates belonging to the class Holothuroidea in Phylum: Echinodermata, inhabit various environments from shallow coastal waters to deep sea. Sea cucumbers perform several significant ecological functions, as efficient ecosystem engineers by deposit feeding habit reduce organic waste, constantly rework the sediments and facilitate bioturbation (Purcell et al., 2016). Sea cucumbers are dried, processed, and sold as a product known as “*Beche- de- mer*” or “*Trepang*”, an expensive Chinese delicacy with growing demand (James and James, 1994). Globally, sea cucumber wild stocks have declined at alarming rates due to overfishing and poor management policies (Conand, 2018). However, aquaculture techniques are well established for commercial species such as *Holothuria scabra* (Giraspy and Grisilda, 2010), which could potentially assist in recovering depleted population and trade in many parts of the world.

Research on sea cucumbers of the Arabian Peninsula (AP) and Iran is scant and sporadic. Hence, a detailed and comprehensive review of the studies conducted in this region is warranted. Habitat diversity and prevailing extreme environmental conditions likely drive sea cucumber diversity in this region; however, such correlations remain poorly studied. In this review we focus on the aspects of historical and current research trends in sea cucumbers of the AP (which includes the following countries: Iraq, Kuwait, Saudi Arabia, Bahrain, Qatar, United Arab Emirates (UAE), Oman, and Yemen) and Iran and their marginal seas (Arabian (AG), Gulf of Oman, Arabian Sea, Gulf of Aden, and Red Sea), to identify gaps in research and provide recommendations to address future research priorities.

### 1.1 Environmental setting and natural history of the Arabian Peninsula

The AP is surrounded by marginal seas including the AG to the east, Gulf of Oman and the Arabian Sea to the south, and the Gulf of Aden and the Red Sea to the west. The APG is a young (<6000 years), shallow sea (average depth 30–60 m), connected to the Gulf of Oman and Indian Ocean by the Strait of Hormuz (Sheppard et al., 2010, Vaughan et al., 2019). The APG has a wide range of sea surface temperature (SST; 12^○^C to >35^○^C) as well as highly variable salinity levels (>42 to >50ppt) (Coles, 2003, Sheppard et al., 2010). The environmental extremes of the AG influences its relatively impoverished species richness and density of this water body (Basson et al., 1977, Jones, 1986, Vaughan et al., 2019). Over 200 species of macroalgae and four species of seagrass (*Halodule uninervis*, *Syringodium istaefolium*, *Halophila ovalis*, and *Halophila stipulacea*) in the AG serve as nursery grounds for several species of invertebrates and vertebrates (Sheppard et al., 2010, Vaughan et al., 2019). Resilient Corals (40 hard coral and 38 soft coral species) and coral reefs thrive in these extreme conditions (Sheppard et al., 2010, Vaughan et al., 2019). However, climate change-induced recurrent bleaching events have led to reef degradation (Sheppard et al., 2010). Furthermore, rapid coastal development in the AP threatens its biodiversity (Burt, 2014).

The Gulf of Oman and the Arabian Sea, which surrounds Oman, consists of deep ocean waters with variable climatic conditions. The southwest summer monsoon triggers upwelling along the coast, which results in movement of the cold, nutrient-rich water to the surface and subsequent increase in primary productivity (Claereboudt, 2019). Rich coral reefs and extensive sandy beaches occur along the Omani coast (Burt et al., 2016, Claereboudt, 2019). In this region, mangrove forests, coral reefs, seagrass, and seaweed communities harbour rich biota (Claereboudt, 2019). During the summer, upwelling leads to low temperature creating a dispersal barrier for the benthic fauna known as a “pseudo-high latitude effect” (Sheppard and Salm, 1988).

The Red Sea is also a relatively young sea, extending >2000 km between the AP and Africa. With an average depth of 500 m, the Red Sea is warm and features relatively elevated salinity levels due to high evaporation rates. Mesoscale eddies play a crucial role in distributing nutrients in the oligotrophic environment of Red Sea (Carvalho et al., 2019). The world’s most productive and biodiverse tropical biotopes including mangroves, seagrass beds, and coral reefs occur in the Red Sea (Carvalho et al., 2019). The coral reefs of the Red Sea accounts for 3.8 % of the world’s reefs, and are rich in diversity of associated fauna (2710 species) with a high degree of endemism (DiBattista et al., 2016, Carvalho et al., 2019). The rich biodiversity of the Red Sea reefs attracts tourists for diving generating massive income and creating opportunities for tourism-based coastal development (e.g., beach resorts). However, such activities pose a serious threat to the biodiversity and functionality of this fragile ecosystem (Carvalho et al., 2019).

## 2 Materials and methods

We categorised research conducted on the sea cucumbers of this region into the following topics:1. Diversity and distribution, 2. Biology, 3. Fisheries, 4. Aquaculture, 5. Ecotoxicology, 6. Bioprospecting, and 7. Other properties. Information on each of this research topic was compiled based on published data (until 31st March 2021) available in the form of peer-reviewed literature (journal articles, books, conference proceedings, etc.). These topics were searched via the Google™ search engine using key words, e.g. “sea cucumber”, “diversity”, “Arabian/ Gulf” for regional and international peer-reviewed publications. We also referred to regional journal databases such as the Scientific Information Database, Iran (https://www.sid.ir/en/journal/) to conduct similar searches. Peer reviewed articles related to aquaculture, fisheries, and diversity were searched in the Pacific Community’s online publication dedicated to international sea cucumber research “Beche-de-mer information bulletin” (https://coastfish.spc.int/en/publications/bulletins/beche-de-mer) using key words denoting a “country” or “marginal sea” of this region. We excluded data from the grey literature due to restrictions in accessibility. For nomenclature and classification, we followed the World Registry of Marine Species (WoRMS) database (WoRMS Editorial Board, 2020)

## 3 Results

### 3.1 Diversity and distribution

**Fig. 1:**
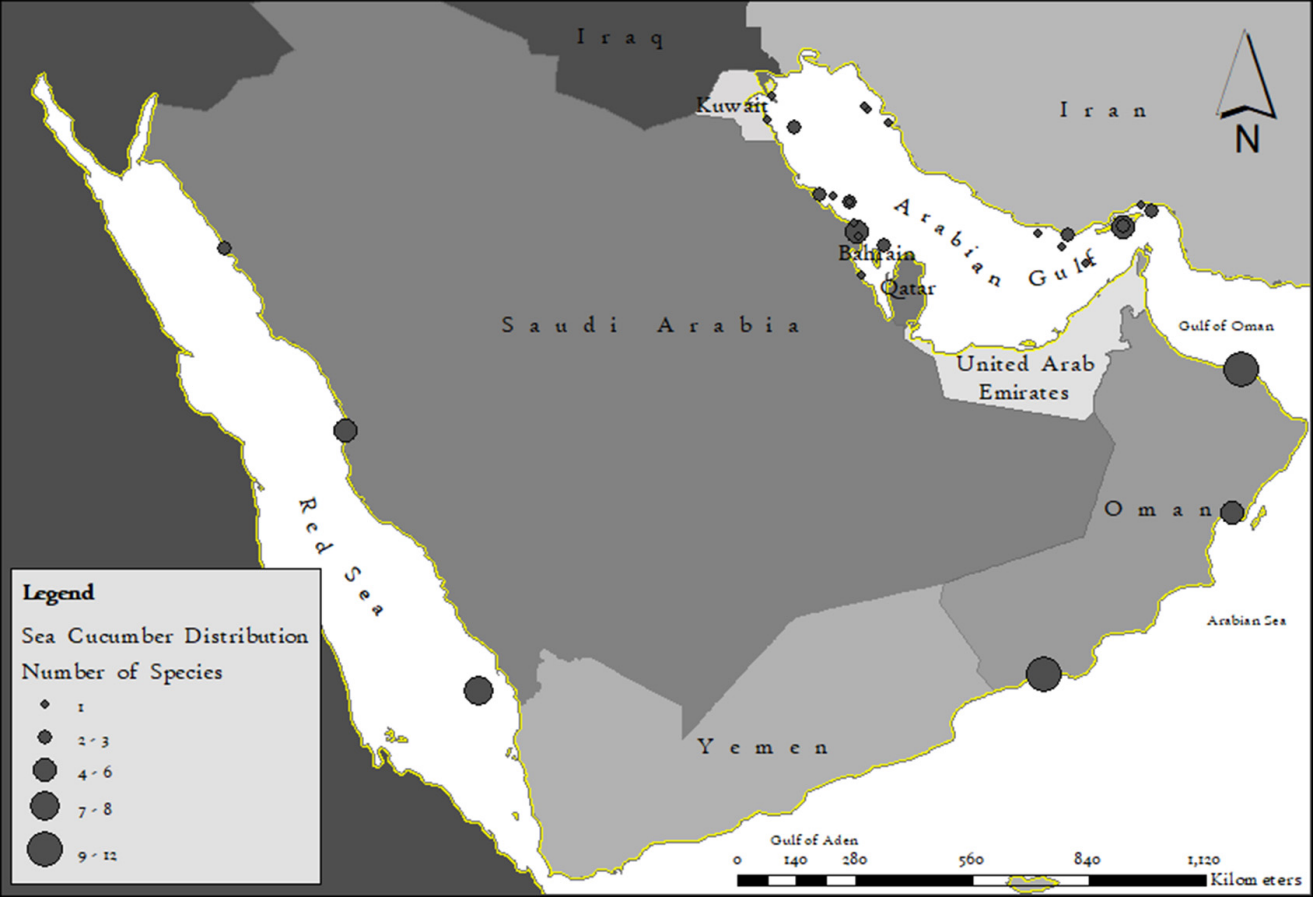
Map of AP and Iran showing the sporadic distribution of sea cucumber species.

**Fig. 2:**
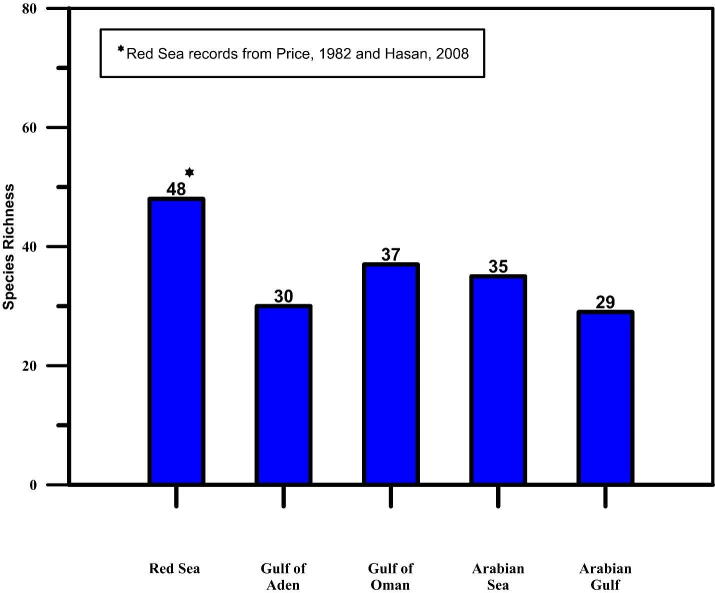
Species richness of sea cucumbers occurring in the marginal seas of the AP and Iran.

Diversity of sea cucumbers in any geographical region serves as a baseline data to stimulate further research and to assist in conservation and management of this invaluable resource. A thorough review of the sea cucumber records from the marginal seas of the AP and Iran indicates a scattered distribution (Fig. 1). An updated checklist of sea cucumber species occurring in the AP and Iran shows a record of 82 species occurring in this region (Supplementary Table S1) and their marginal seas (Fig. 2) indicates impoverished diversity in the region with need for investigation in deeper waters.

Historical investigations related to sea cucumbers in the AP and Iran date back to the *“RIMS Investigator*” collections of Koehler and Vaney (1908). They recorded five species: *Halodeima parva*, *Holothuria monocaria, H. vagabunda (=leucospilota), H. ocellata, and Stichopus variegatus (=hermannii)* from Bushehr province and Bandar Abbas, in the AG and one species, *Thyone festina*, from Northeast of Bahrain. Thirty-two years later, a Danish expedition was conducted in the AG during which extensive sampling was carried out in 156 locations along the coast of Iran and around Bahrain (Hedging, 1940). This was the most extensive collection of sea cucumbers carried out in this region, and a total of 17 species were recorded, adding two new species to science. Hedging (1940) also indicated that sea cucumbers recorded in the AG have a high degree of endemism. Later, Clarke and Rowe (1971) in their monograph on Indo–West Pacific echinoderms reported the diversity and distribution of sea cucumbers from this region. Zoogeographic relationships of sea cucumbers in this marginal environment is very important to understand similarities in species composition and endemism. On a dedicated study related to the zoogeography of echinoderms of AP, Price (1982a) reviewed the records of echinoderms from the marginal seas of the AP to analyse species similarity and endemism. He observed high species diversity in the Red Sea, similarity of AG fauna with Red Sea, South East Arabia (Gulf of Aden and Arabian Sea) and also a high degree of endemism in SE Arabian waters (Gulf of Aden and Arabian Sea) due to its connectivity with the Indian Ocean.

Recently, several studies have focused on documenting the diversity and distribution of sea cucumbers occurring in the AP and Iran (Table 1). The recent record of *H. scabra* from Sharjah, UAE (Yaghmour and Whittington-Jones, 2018) presents strong incentive to explore the extent of regional distribution of a commercially important species and investigate prospects for aquaculture to conserve stocks and revive trade. Apparently, majority of studies included in this review are limited to shallow habitats (0–20 m depth), except for the study by Price (1982). Hence, the species composition in the deeper habitats of Red Sea and Oman could certainly reveal the underestimated diversity and actual distribution patterns. Like any other fauna, the environmental extremes of this region potentially contribute to the impoverished diversity of the sea cucumbers in this region, yet this is also poorly understood.

**Table 1:** Recent studies related to diversity and distribution of sea cucumbers in AP countries and Iran.

Species	Location	Marginal Sea	Details	References
*Holothuria atra*	Tarut Bay, Saudi Arabia	AG	Studies on the biotopes of western AG	Basson et al. (1977)
Thirteen species of Holthuroidea	Saudi Arabia	AG	Studies on the echinoderm diversity and distribution in the Arabian Gulf coast of Saudi Arabia	Price, 1981, Price, 1983
Eighty three species of Holothuroidea	AP countries and Iran	AG, Gulf of Oman, Arabian Sea, Gulf of Aden and Red Sea.	Biogeography of the echinoderms of Arabian Peninsula.	Price (1982a)
*H. arenicola* & *Stichopus hermanni*	Kuwait	AG	Intertidal fauna along the Kuwaiti coast.	Jones (1986)
*Oshimella ehrenbergii*	Kuwait Bay, Kuwait	AG	Winter species composition of benthic fauna in Kuwait bay.	Al-Yamani et al. (2009)
*H. arenicola*	Al Zour, Kuwait	AG	Intertidal collection	Deemer (2011)
Holothuroidea	Kuwait Bay, Kuwait	AG	Investigation on benthic community structure of Kuwait bay indicated the occurrence of one unidentified species of Holothuroidea	Al-Rifaei et al. (2012)
*H. atra*, *H. arenicola* and *O. ehrenbergii*	Kuwait Bay	AG	Yearlong study on the community structure of zoobenthos in Kuwait Bay. Record of *H. atra* is a misidentification and it is probably a colour morph of *H. arenicola,* needs molecular investigation.	Al-Yamani et al. (2012)
*H. hilla* & *H. parva*	Chabahar Bay, Iran	Gulf of Oman	Ecological survey of sea cucumbers at 5m depth with habitat characteristics	Shakouri et al. (2009a)
*H. leucospilota*, *H. arenicola and Stichopus variegatus* (*=hermanni*)*.*	Chabahar Bay, Iran	Gulf of Oman	Ecological survey of sea cucumbers occurring in sand-mud and rock substrates.	Shakouri et al. (2009b)
Twenty four species of sea cucumbers.	Oman	Arabian Sea & Gulf of Oman	Inventory of sea cucumbers species of Omani waters.	Claereboudt and Al-Rashdi (2011)
*H. insignis*	Chabahar Bay, Iran	Gulf of Oman	Intertidal quantitative survey of sea cucumbers indicated high frequency of occurrence during January 2009 and random distribution during the survey period.	Matin et al. (2011)
*H. arenicola*	Chabahar Bay, Iran	Gulf of Oman	Transect surveys shows that *H. arenicola* is usually rare and abundant during January 2009.	Matin (2018)
*H. parva, H. arenicola, cinerascens, H. hilla, H. leucospilota* and *H. pardalis*	Qeshm Island, Iran	AG	Intertidal survey of sea cucumbers.	Reza et al. (2011)
*S. hermanni (=S. variegatus*)	Kish Island, Iran	AG	Occurrence of *H. heramanni* in the reef area with detailed morphological description	Tehranifard et al. (2011).
*H. hilla*	Farur Island, Iran	AG	Recorded at 14m depth by SCUBA diving.	Dabbagh and Kamrani (2011).
*H. arenicola, H. parva* and *H. leucospilota*	Bandar -e- Bostaneh, Iran	AG	Intertidal survey	Dabbagh and Keshavarz (2011)
*O. ehrenbergii*	Farur Island, Iran	AG	Description with habitat characteristics.	Dabbagh et al. (2012b)
*H. scabra*	Qeshm Island, Iran	AG	Collected at 5 m depth by snorkeling. Commercially important and a new record to AG	Dabbagh et al. (2012c)
*H. hilla, H. impatiens, H. leucospilota, S. hermanni and S. monotiberculatus*	Larak Island, Iran	AG	Subtidal survey	Khazaali et al. (2012)
*H. leucopsilota* and *S. hermanni*	Abu Musa Island, Iran	AG	Intertidal survey	Majid et al. (2012)
*S.*cf. *monotuberculatus*	Hengam Island, Iran	AG	Intertidal survey	Maryam et al. (2012)
*H. hilla, H. impatiens, H. leucospilota, H. scabra, S. hermanni and S. monotuberculatus*	Hengam Island, Iran	AG	Underwater survey by SCUBA diving at 5-15m depth	Salarzadeh et al. (2013)
*H. hilla, H. leucospilota, H.* *impatiens* and *S.hermanni*	Hendourabi Island, Iran	AG	*H. leucospilota* and *S. hermmanni* were abundant	Ameri et al. (2014)
*H. parva*	Bostaneh Port, Iran	AG	Morphology and molecular phylogeny.	Eshanpour et al. (2016)
*Protankyra pseudodigitata*	Bushehr Port, Iran	AG	Subtidal collection at 8.5 m depth using a Van Veen grab.	Peyghan et al. (2018)
*H. scabra*	Alqurm Wa Lehhfaiiah Protected Area, UAE	Gulf of Oman	Collected from sandy and seagrass beds during low tide.	Yaghmour and Whittington-Jones (2018)
*H. arenicola, H. parva, S. horrens* and *S. monotuberculatus*	Bastaneh Port and Hendourabi Island, Iran	AG	Tidal zone survey of sea cucumbers.	Noura et al. (2019)

### 3.2 Repetitive records

Recent records of sea cucumbers from the coast of Iran are repetitive in nature and carry distribution information of a species previously recorded from the APG. Such redundancy in citing these records could only create confusion. E.g. Pourvali et al. (2014) surveyed shallow water sea cucumbers from Hormuz Island near the Strait of Hormuz and recorded six species of sea cucumbers. However, they report that *H. bacilli* (see Tables 2 and 3, p. 398) as an additional record, without an illustration. Later, Afkhami et al. (2015) recorded two species of sea cucumbers, *H. bacilli* and *H. insignis*, as new records to the APG from Hormuz Island, among which the former species is already reported as “common” by Pourvali et al. (2014) from the same location. Therefore, Afkhami et al. (2015) cannot be treated as a new record. Likewise, it is important to note that there are similar reports from the waters of Iran Dabbagh and Kamrani, 2011, Dabbagh and Keshavarz, 2011 of species already known from this region. Distribution records for any species are helpful to understand their distribution range, however repetitive records as shown here could only create more chaos in the literature. Exclusion of such publications for any kind of biodiversity or biogeography related data analysis is essential. We solely intend to point out at these records as an attempt to bring clarity in the literature.

## 4 Biology

Scientific data related to the biology of sea cucumbers is crucial in framing fisheries and conservation management policies. Studies related to sea cucumber biology were largely reported form the coast of Iran and ideally focused on reproductive biology. A detailed 16-month investigation of the reproductive biology of *S. hermanni* population in Kish Island, Iran shows that peak spawning occurs during the summer months (July-August) and gametogenesis occurs in the spring (March) (Tehranifard et al., 2006, Tehranifard et al., 2007). Though *H. leucospilota* is a low value species, understanding its reproductive biology could help in developing viable culture techniques. Five stages of gonadial development were identified in male and female *H. leucopsilota* collected from Bostaneh coast, Iran (Morovvati et al., 2011, Ghobadyan et al., 2012). In the southern Iran population mean size recorded at sexual maturity was 246 mm (male) and 220 mm (female) respectively (Seyed et al., 2020. Two peak spawning seasons were identified, with larger peak occurring in June and July and smaller peak in December and January (Seyed et al., 2020).

The majority of commercially important sea cucumber species have active deposit and suspension feeding habits (Purcell et al., 2016). However, very little information exists on feeding biology of sea cucumbers in the AP and Iran. In Iran, dietary habits of *H. scabra* were studied for the population thriving in Qeshm island. Gut content analysis indicated sludge was the major source of food followed by black sandy grit and crustacean exoskeleton along with green algae, invertebrates, etc., (Rezvani and Mohammadizadeh, 2014). Anatomical investigations could help in understanding evolutionary mechanisms involved in adapting to extreme environmental conditions. Comparative histological investigations of the digestive tract and skin of sea cucumbers (both high and low value species) provide valuable insights into the anatomical features of these ecologically valuable invertebrates (Pouria et al., 2014, Pouria et al., 2015).

The AG, due to its high salinity (>42 PSU) and wide temperature range (12–33 °C) poses physiological challenges for organisms that must adapt to thrive in extreme conditions. A high range of tolerance to salinity observed in echinoderms of AG waters indicates *H. leucospilota* and *Leptosynapta chela* can thrive at salinities up to 43‰ and 52–55‰, respectively (Price, 1982b). The tolerance of sea cucumbers to hypersaline conditions prevailing in these marginal seas requires further investigation and large knowledge gap exists in understanding their survival thresholds

Furthermore, aspects of the biology of sea cucumbers along the Red Sea coast of Saudi Arabia and Yemen are unknown. Due to its immense fishery value, investigations on the reproductive biology of *H. scabra* were carried out along the Omani coast (Al-Rashdi and Claereboudt, 2018). The outcomes of these investigations indicate that the spawning season coincides with the onset of increasing temperatures during the summer months (Al-Rashdi and Claereboudt, 2018). As far as biology of sea cucumbers are concerned, an ample scope for regional research exists to understand physiological impacts of natural and anthropogenic stressors, food preferences, nutrition, diseases, etc.,

## 5 Fisheries

On a global scale, artisanal sea cucumber fisheries have declined in many countries due to over exploitation (Purcell et al., 2013). Recent addition of contributing countries has led to an increase in the fisheries stocks to over 16, 000 tons/year (Conand, 2018). Commercially important species such as *H. scabra* is largely an artisanal fishery resource and populations are mostly restricted to shallow sandy habitats. In the AP, small scale artisanal fisheries exists in Saudi Arabia, UAE, Oman, and Yemen. Recently, a dedicated fishery for sea cucumbers was identified in Qeshm Island, Iran following interviews with local fishermen (Afkhami et al., 2012). Baseline information on various aspects of sea cucumber fisheries exists for the AP and Iran (Table 2). There is no documented evidence of existing sea cucumber fisheries along the eastern coast of the AP due to lack of high value species such as *H. scabra*, *H. nobilis*, etc. However recently, *H. scabra* was recorded from Gulf of Oman coast of UAE (Yaghmour and Whittington-Jones, 2018), but strict management actions would be necessary to conserve this population. In the future, intensive surveys along the east coast of the AP are essential to investigate the distribution range of commercially valuable species and their population characteristics.

**Table 2:** Baseline information pertaining to sea cucumber fisheries of the AP and Iran.

Location	Topic	Description	Source
Yemen	Fishery	• Abundant species-*H. scabra*, collected by skin diving • Sea cucumbers from the Red Sea and East Africa are received in Yemen and exported to Singapore. • Catch declined from 200 to 30 t.yr^−1^ (1970–1985).	Amir (1985)
Mahout Bay, Oman	Density, size distribution and fishery	• Minor fishery exists for *H. scabra* in Mahout bay from 1960 onwards, with women dominating (50%) the fishing group. • Population surveys (2004–2005): population density-1170 and 4000 kg. h^−1^_,_ biomass- 393 and 2903 kg.h^-1^and sex ratio – 1:1 • *H. scabra* is collected from six sites during spring low tides, dried, processed as *Beche-de-mer* and exported through UAE fetching a price of 35–55 OMR (Omani Riyal). kg^−1^	Al-Rashdi et al., 2007a, Al-Rashdi et al., 2007b
Wajh, Thuwal and Farasan Islands, Red Sea, Saudi Arabia	Fishery status and management plan	• Catch Per Unit Effort (CPUE) declined from 126.9 to 6.3 (Kg^-1^fisher^-1^day^−1^) during 2000–2003. • Overfishing, drastic decline in landings from 1, 997 (1999) to 230 (2004) tonnes.	Hasan (2008)
Wajh, Thuwal and Farasan Islands, Red Sea, Saudi Arabia	Stock assessment	• Surveys conducted in 18 sites, 12 species identified. • Commercial species are overfished due to lack of management.	Hasan (2009)
Mahout Bay, Oman	Over fishing	• Artisanal fisheries of sea cucumbers in Mahout bay (320 km^2^). • In 2005 estimated biomass was 1500 tonnes • Processed *H. scabra* exported through UAE – 14.5 tonnes • During 2005–2008 decline in sea cucumber density observed, • Demand for *H. scabra* led to depletion of stocks, target fishery shifted to less valuable species (*H. atra* and *H. leucospilota*). • Harvest of immature individuals < 12 cm (fresh) and < 6 cm (dried) for processing. • Ministry of fisheries formulated management interventions: fishing size regulation (min 20 cm), seasonal fishing closure (February-August) and aquaculture to sustain fishermen livelihood.	Al-Rashdi and Claereboudt (2010)
Qeshm Island, Iran	Fisheries	• Two year study (2004–2006) on sea cucumber fisheries indicates increase in number of fishers and density upto 30 ind.h^−1^	Afkhami et al. (2012)
Qeshm Island, Iran	Fisheries	• During 2004–2006, *H. scabra* was collected by 150–200 fishermen from seven fishing sites at 15–18 m by SCUBA diving. • Fishing trip – 5–6 h.d^-1^, 150–200 live animals were collected. • Suggested management interventions- studies on ecology, biology and ban on fishing is recommended to revive stocks.	Afkhami et al. (2013)
Al Wusta Governorate, Oman	Small scale fisheries contributing to the livelihood of fisherwomen	• Women contribute to small scale invertebrate fisheries. • Women are actively involved in collecting *H, scabra* and the catch is sold to a trader.	Al-Rashdi and McLean (2014)
Mahout Bay, Oman	Fishery and biology	• Catch of *H. scabra* increased from 3.6 t (2013) to 39.4 t (2015), juveniles targeted. • One year (2018–2019) fishing ban introduced by the government extended upto March 2021. • Length-weight relationship -females longer than males and the sex ratio is 0.49.	Al-Jufaili et al. (2021)

**Fig. 3:**
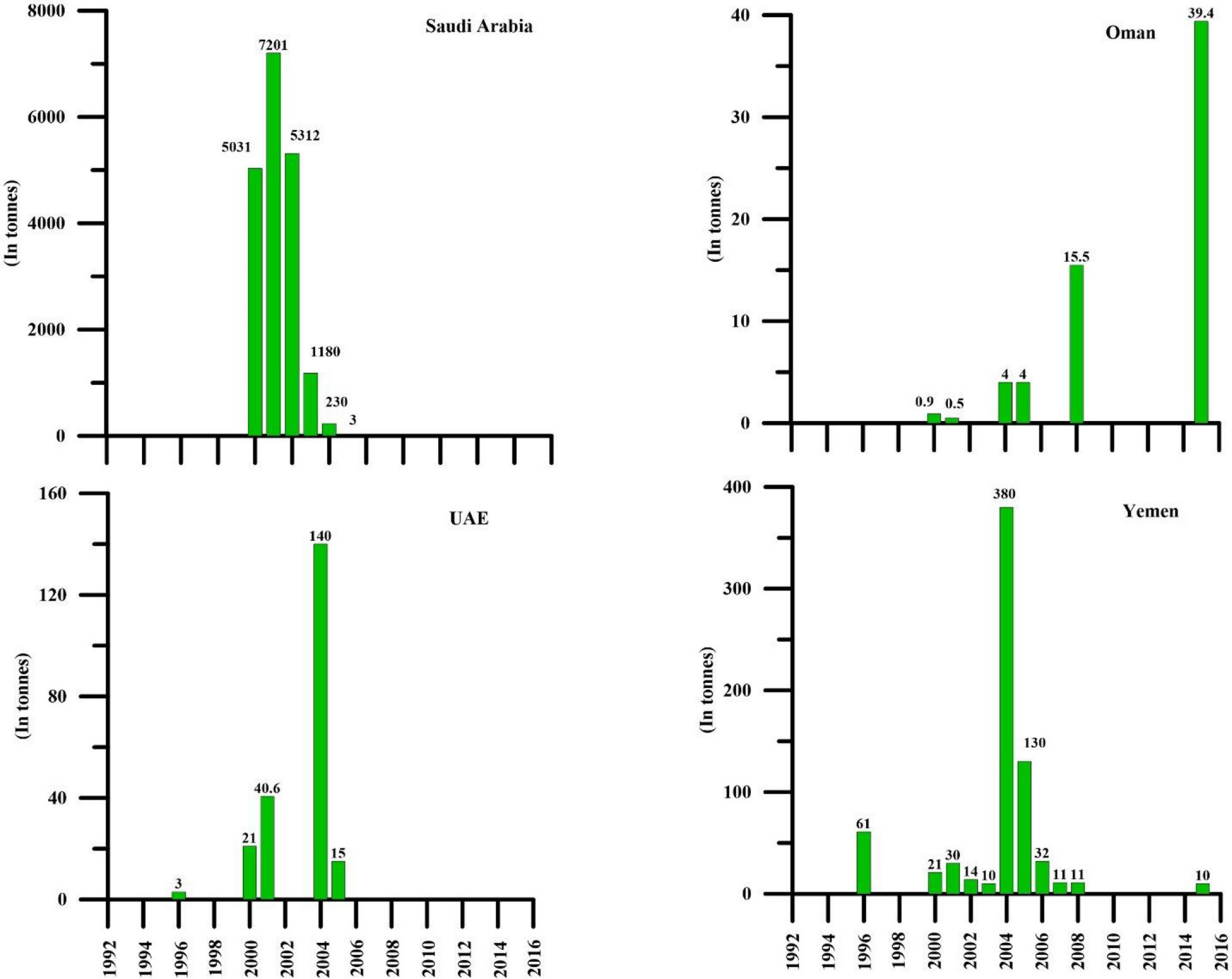
Annual export of dried sea cucumbers (in tonnes) from the AP countries to Asian markets during 1996–2014 (data compiled from Purcell et al., 2013, Muthiga and Conand, 2013, Conand, 2018).

Published datasets (1996–2014) indicate that the aforementioned AP countries are the major players in collecting and exporting dried sea cucumbers through the UAE to the Asian markets (Purcell et al., 2013, Muthiga and Conand, 2013, Conand, 2018). The data on export of dried sea cucumbers from the AP shows a steady decline (Fig. 3). Exports from Saudi Arabia to Asian countries have significantly reduced due to depleted stocks (Hasan, 2008). Yemen, a majorexporter, experienced drastic decline in sea cucumber exports from 60 to 21 tons. yr^−1^ year between 1986 and 2014 (Conand, 2018). In the case of Oman, exports were reduced due to overfished stocks (Al-Rashdi and Claereboudt, 2010). In UAE, although no dedicated fishery for sea cucumber is known, yet decline in exports were necessarily due to low supply of dried material. Among the AP countries involved in exporting dried sea cucumbers to the Asian markets, the UAE plays a pivotal role as a collection centre and effectively carries out all export operations due to the availability of excellent infrastructure facilities.

Unregulated fishing practices have already taken a toll on sea cucumber fisheries worldwide (Purcell et al., 2013). Research on the aspects of sea cucumber fisheries in the AP and Iran (Table 2) indicates a drastic decline in stocks, which calls for robust management practices to be adopted to recuperate the damaged stocks. On a global scale, effective management strategies are already adopted by several sea cucumber fishing nations (Purcell et al., 2012). In order to manage endangered sea cucumber populations along the Red Sea coast of Saudi Arabia, a stringent management plan was suggested (Hasan, 2008). Networking among the sea cucumber fishing countries in the AP and Iran could facilitate exchange of suitable information to frame effective management policies and guidelines to manage this fragile fishery resource. Recent advances in aquaculture techniques and availability of culture technologies for many commercially valuable species of sea cucumber (E.g. *H. scabra* and *H. lessoni*) could effectively assist in reviving damaged stocks in the wild and in recovering fisheries and trade (Giraspy and Grisilda, 2010).

## 6 Aquaculture

Large scale aquaculture programs for sea cucumber can be a fruitful solution to conserve depleted stocks through larvae/juvenile ranching. Also, commercial production can supply material to meet the growing demand for *beche- de- mer* in the international market. Standardized commercial hatchery technology for high value species such as *H. scabra* is already available (James et al., 1994, James, 2004, Giraspy and Grisilda, 2005). Iran has bright prospects for sea cucumber aquaculture with the availability of *H. scabra* stocks in the wild (Afkhami et al., 2013). The developmental stages of sea cucumber include: a) Auricularia (larval phase), b) Doliolaria (larval phase) and c) Pentactula (settlement phase). These are followed by metamorphosis into a juvenile (James, 2004). Larval rearing techniques for commercial species such as *H. scabra* are well established (Giraspy and Grisilda, 2005). Recently, successful breeding and larval rearing of both low (*H. leucospilota*) and high value (*H. scabra*) species of sea cucumbers were accomplished in Iran (Dabbagh et al., 2011a, Dabbagh et al., 2012a).

In 2011, Iran succeeded in induced breeding and larval rearing of *H. scabra* and produced hatchery reared individuals up to 22 g in weight after one year (Dabbagh and Sedaghat, 2012). However, slow growth rate was achieved in juveniles compared to what is achieved for the same species elsewhere (Battaglene et al., 1999, James, 2004). Slow growth rate witnessed in cultured *H. scabra* juveniles in Iran is possibly an impact of high-water temperature (>30 °C) or salinity in the AG, as witnessed earlier for *H. leucospilota* (Dabbagh et al., 2011a). In the case of *H. leucospilota*, a low survival rate of 4.2 % was achieved in juveniles after successful induced spawning by thermal shock and by combining both water pressure and thermal shock methods (Dabbagh et al., 2011a). The survival rate of Dolioalria larvae was only 40 % in 22 days, and again higher salinity possibly impacted growth rates. More dedicated research is essential in this direction to identify the factors influencing growth rate of sea cucumber juveniles for profitable aquaculture ventures.

During the larval rearing of *H. scabra*, larvae settlement on suitable growth substrates dictates the success of survival and metamorphosis of early Auricularia larvae into Pentactula and juvenile stages. Dabbagh et al. (2011b) experimented on *H. scabra* larval settlement on various substrates including plastic sheets, rough tile surfaces, and 500 µm plankton nets, and it was observed that the Pentactula larvae had a higher preference for settlement on 500 µm plankton net compared to the other substrates used in the experiment. In terms of juvenile *H. scabra*, use of *Sargassum* and *Padina* powder mixed with fine sand and coral substrates resulted in increased good growth rate compared to the use of a substrate with *Sargassum* powder and fine sand mixture (Esmail Zade et al., 2012).

Another key aspect in sea cucumber culture is identifying the appropriate food for larval rearing, as it plays a vital role during various stages of larval development (Giraspy and Grisilda, 2005). Recently, Dabbagh et al. (2012a) experimented on the effects of commercial feeds on *H. scabra* and observed that coating of settlement plates with Algamac protein induced larval settlement and growth. Whereas in the case of juvenile *H. scabra*, feeding with shrimp starter food showed 25% increase in protein and increased survival rates (Noverian and Biabani, 2018). In addition to monoculture studies on *H. scabra*, experimental trials to culture shrimp (*Penaeus indicus*) and other sea cucumber species (*H*. *leucopsilota*) were also successfully conducted in Iran (Amini Rad, 2005).

Due to the availability of established *H. scabra* population in the region, small to large scale aquaculture ventures are possibility. Recently, the National Prawn Company, Saudi Arabia, the largest aquaculture enterprise in the region, has successfully bred *H. scabra* and given some promising projections for export to meet the ever increasing demand in the Asian markets and to recover depleted stocks along the Red Sea coast of Saudi Arabia (Anonymous, 2019). Along with fin fish cage culture in Saudi Arabia, sea cucumbers can be cultured below these cages so that a constant supply of nutritious food will be available from the cages (Cardia et al., 2015).

Prospects for sea cucumber aquaculture are also being explored in Oman (FAO, 2010), where available stocks of *H. scabra* have undergone a drastic decline due to overfishing (Al-Rashdi and Claereboudt, 2010). Thus, promising hatchery trials were conducted by inducing wild caught individuals to spawn through the “Maturation Inducing Fractions (MIF)” method, and detailed methodologies for successful hatchery development are available for students, technicians, and entrepreneurs (Al-Rashdi et al., 2012, Al-Rashdi et al., 2018). An impressive 90 % fertilization rate was achieved, with 9% post-fertilization larval (Pentactula) settlement (Al-Rashdi et al., 2018). However, the initial mortality rate was around 70% during the early development phase due to ciliate protozoans and copepod infestations. Detailed investigations have to be carried out to curtail early larval mortality by improvising water quality and filtration methods.

Achieving success in breeding and larval rearing of high value species such as *H. scabra* in this region can be a promising aquaculture venture. So far, results obtained from Oman and Iran on *H. scabra* breeding and larval rearing is very encouraging for promoting sea cucumber aquaculture experiments in the region. Such endeavors offer prospects for large-scale commercial sea cucumber farming in the AP, which can aid in recuperating damaged wild stocks and can also contribute to regional economic growth.

## 7 Ecotoxicology

The post-gulf war economic boom led to intense population growth, rapid coastal development, new desalination plants, and increased sewage and industrial waste disposable, which collectively contributed to the environmental degradation of the region (Sale et al., 2011, Sheppard et al., 2010). The Gulf war oil spill had devastating environmental impacts on this fragile and unique ecosystem, with hydrocarbon contamination reaching up to 400 km offshore (Fowler et al., 1993, Sale et al., 2011, Sheppard et al., 2010). Moreover, this region has the world’s highest oil tanker traffic and the combustion through oil production, vehicles, atmospheric transport, and other activities serve as a source of polyaromatic hydrocarbon (PAH) contamination in both sediment and biota (Khazaali et al., 2016, Sale et al., 2011).

Ecotoxicological investigations on sea cucumbers as bioindicators of environmental contamination is rather limited in the AP and Iran. Thus, vast research opportunities exists in this anthropogenically impacted region to understand the impacts of pollution on this marginal environment and to assist stake holders in framing management guidelines and policies. Recently, the impact of PAH on the composition of essential fatty acids (EFA), which plays a crucial role in the metabolism of sea cucumbers including *H. leucospilota* and *H. scabra*, was investigated in Iran (Kamyab et al., 2016). Variable EFA concentration in tissue samples taken from animals from polluted versus non-polluted sites indicates that the PAH concentration may have potential negative impacts on benthic fauna (Kamyab et al., 2016). Sea cucumber mortalities due to natural events (e.g., algal blooms) and contaminants are rare in this region. However, a recent incident of mass mortality of *H. arenicola*, possibly due to osmotic shock caused by storm water drainage, was recorded from Kuwaiti shores (Al-Yamani et al., 2020).

Some heavy metals play a crucial role in organismal metabolism (e.g., enzymatic pathways), while certain other metals such as Mercury (Hg) are extremely toxic and could enter into the food chain through various routes of contamination. Sea cucumbers serve as valuable bioindicators of metal contamination in diverse habitats (Warnau et al., 2006, Culha et al., 2016, Ahmed et al., 2017). Along the coast of Iran, concentrations of heavy metals (Zinc (Zn), Cadmium (Cd), Copper (Cu), and Lead (Pb)) was higher in the skin, gonads, and respiratory trees of *H. leucospilota* species, whereas in *H. scabra* heavy metal concentrations were higher in gonads and the dermal layer (Mohammadizadeh et al., 2015). Furthermore, this study shows that the concentration of Cd and Pb exceeded the lower prescribed limit for human consumption, whereas levels of Cu and Zn were within the accepted limit for human consumption. It is essential to enforce stringent testing protocols for these contaminants prior to processing of sea cucumbers from this region for export to Asian markets.

Recently, microplastics were identified as a global threat by entering into the ocean’s food chain. Ingestion of microplastics by marine organisms can lead to their subsequent transport into the human food chain due to seafood consumption. Holothurians, being benthic feeders, could potentially ingest plastic in various forms including phthalate esters. Phthalate concentration in *H. atra* from Iran was found to be at a high level. This finding is indicative of environmental contamination due to plastics and it also demonstrates the value of using sea cucumbers as bio-marker for pollutant detection (Sharifi et al., 2017). Several recent studies have addressed microplastics pollution in the AG (Al-Salem et al., 2020a, Al-Salem et al., 2020b: Saeed et al., 2020, Uddin et al., 2020,), and the findings from these studies invite further research on microplastics and their impacts on benthic deposit feeding invertebrates like sea cucumbers.

Sea cucumbers also a play vital role in reducing organic pollution and recycling of nutrients through their deposit feeding habits (Uthicke, 2001, Slater and Carton, 2009). Recent lab-based experimental trials conducted in the Kuwait Institute for Scientific Research using *H. atra* and *S. hermanni* also indicates their potential utility in bioremediation of coastal habitats from rising organic pollution and their capacity to reduce nutrient loading in aquaculture systems (Al-Yaqout et al., 2017).

## 8 Bioprospecting

Screening bioactive substances from marine organisms has been very promising in recent years due to immense potential for discovery of compounds with nutritional and pharmaceutical value (Bordbar et al., 2011, Lindequist, 2016). Bioactive compounds identified from sea cucumbers have strong anti-microbial properties (Kiani et al., 2014), which gave the impetus to the global research community to identify novel compounds for drug development. Sea cucumbers in the AG thrive in extreme environmental conditions (high salinity and temperature) and their active metabolites may likely prove valuable with very high pharmacological potential (Khotimchenko, 2018, Dakrory et al., 2015, Diba et al., 2017). Due to high diversity and abundance of sea cucumbers occurring along the coast of Iran, focus on bioprospecting research increased in the recent years with several promising results (Supplementary Table S2).

## 9 Other properties

In addition to routine bioprospecting investigations, sea cucumbers have also been shown to possess intriguing and unique properties. For example, extracts of *Holothuria parva* have been shown to reduce the impact of electromagnetic radiation on the testes of male Balb/C mice (Vasei et al., 2015). Recently the anti-fouling and anti-microbial potential of *H. leucopsilota* was investigated, and fatty acids and Terpenes identified in ethyl acetate extracts of the body wall showed strong activity against Barnacle larvae and *Staphylococcus aureus* (Darya et al., 2020).

Enzyme inhibitors and antioxidants play a crucial role in treating type II Diabetes. Screening of ethanolic extracts of respiratory trees of *S. hermanni* and *H. leucospilota* collected from Iran showed a very high antioxidant activity and inhibited the β-glucosidase enzyme (Abbasi et al., 2019). *In vitro* investigations on the effect of methanol extracts from *H. leucospilota* on *Leishmania major*, an endoparasite responsible for the tropical disease “Leishmaniasis”, proved fatal for its promastigotes (Foroutan-Rad et al., 2016).

Sea cucumbers also possess very promising medicinal properties which could help in developing valuable therapeutics to solve a range of medical conditions. Sea cucumbers from Iran have been the subject of various reproductive endocrinology research activities (Golestani et al., 2016, Moghadam et al., 2016). Methanol extracts of *H. leucospilota* promote oocyte maturation a in rats and further studies could lead to development of novel therapeutics to treat infertility (Khalizadeh et al., 2016). Furthermore, peptides hydrolyzed from the muscles of AG sea cucumbers possess anticoagulant properties similar to Heparin (Samira and Saber, 2017).

## 10 Conclusions

This review examined historical and currents research trends in sea cucumbers of the AP and Iran in order to identify lacunae in knowledge and recommend strategies for future research. Based on the literature records 82 species are known from this region (Table 1), yet it is important to extend investigations into deeper waters (>100 m) to reveal the actual species richness from the marginal seas of this region. Being one of the world’s oil rich region with the highest cargo vessel traffic, investigation on possible introduction of alien species of sea cucumbers from ballast waters can be another interesting avenue in documenting biodiversity research.

Phylogenetics and evolutionary mechanisms in these environmental extremes offers promising research opportunities. An integrated approach through molecular taxonomy can resolve problems in sea cucumber taxonomy and can also help in the process of identifying illegally exported material from this region. Similarly, investigations on the population genetics of sea cucumbers in this region are rare. Such investigations will aid in understanding endemism and identifying any genetic exchanges that may occur between neighboring populations in the Indo-Pacific.

Knowledge gaps exist in understanding the biology of sea cucumbers of commercial value in this region. Also, research related to sea cucumber biology needs to focus on a broader perspective to include both commercial and non-commercial species. The extreme physical conditions of the AG (Sheppard et al., 2010) tests the physiological limits of organisms living in this marginal environment. Hence a thorough understanding of the biology of sea cucumber species in this region could broaden our understanding of their adaptability to events induced by climate change and could also assist in development of effective management plans for overfished stocks.

Systematic stock assessment surveys are essential to manage overfished sea cucumber stocks in this region. Further investigations along the Red Sea coast of Saudi Arabia and Yemen are necessary to have more clarity in fisheries data. Management plans to recover sea cucumber stocks impacted by overfishing require stringent action (Hasan, 2008). Banning sea cucumber fishing could seriously impact the livelihood of artisanal fishing communities, hence introduction of alternate livelihood options is required to alleviate poverty and should be an integral part of the management strategy.

Breeding and larval rearing techniques have been well established for commercially important species such as *H. scabra* (James, 2004), hence aquaculture can potentially revive damaged wild stocks of this species through ranching and can also supply processed material for export to Asian markets. Involving sea cucumber fishing communities by providing appropriate training in aquaculture techniques could provide alternate livelihood options. In recent years, *H. scabra* culture techniques have advanced by improvising larval feed thus leading to increase in growth and survival rates (Giraspy and Grisilda, 2010). Iran, Saudi Arabia, and Oman have succeeded in culturing *H. scabra* (Dabbagh et al., 2011a, Cardia et al., 2015, Al-Rashdi et al., 2012)*.* Thus, expertise from these countries can be used to offer regional training and capacity building opportunities, which can assist in disseminating the relevant knowledge to other countries in the region.

Sea cucumbers function as biomarkers for pollution. They constantly feed on sediment organic matter making them viable candidates for bioremediation of polluted sites. The extreme environmental conditions of the AP could lead to the production of an interesting combination of bioactive compounds in sea cucumbers. Iran leads in regional sea cucumber bioprospecting research (Supplementary Table S2). Further research on characterizing the compounds could aid in developing drugs against deadly diseases such as cancer that threaten mankind.

This review reveals that sea cucumbers of the AP and Iran are not only a valuable ecological entity, but also a potential fishery resource and a valuable candidate for aquaculture capable of generating huge revenue through export. The available funds and sound scientific infrastructure in this region have to be utilized effectively through regional co-operation and networking to foster further research. Unaddressed research topics identified through this review could stimulate multidisciplinary research and assist in the conservation and management of this indispensable resource.

## Declaration of Competing Interest

The authors declare that they have no known competing financial interests or personal relationships that could have appeared to influence the work reported in this paper.
