# Supplementary material for: Sea cucumbers of the Arabian Peninsula and Iran – A review of historical and current research trends
Source: Saudi J Biol Sci. 2021 Oct 6;28(11):6116–26. doi: 10.1016/j.sjbs.2021.10.001 (PMC8568818; doi:10.1016/j.sjbs.2021.10.001)
Supplement: Supplementary data 1 [file mmc1.docx]

Table S1. Distribution of sea cucumbers in the AP and Iran (1=Iraq, 2= Kuwait,

3=Bahrain, 4=Saudi Arabia, 5=Qatar, 6=UAE, 7= Oman, 8= Yemen, and 9= Iran). Classification is based on WoRMS database (Horton et al., 2021)

| Species | Synonym | 1 | 2 | 3 | 4 | 5 | 6 | 7 | 8 | 9 | Remarks | Source |
| --- | --- | --- | --- | --- | --- | --- | --- | --- | --- | --- | --- | --- |
| **Dendrochirotida Grube, 1840** |  |  |  |  |  |  |  |  |  |  |  |  |
| **Cucumariidae Ludwig, 1894** |  |  |  |  |  |  |  |  |  |  |  |  |
| *Leptopentacta imbricata* (Semper, 1867) | *Oncus javanicus* Sluiter, 1880 |  |  |  |  |  |  | + | + | + |  | Price, 1982a |
| *Plesiocolochirus armatus (*von Marenzeller, 1882*)* | *Colochirus loeppenthini* Heding, 1940 |  |  | **+** |  |  |  |  |  | + |  | Heding, 1940; Dabbagh et al., 2012b |
| *Plesiocolochirus dispar* (Lampert, 1869) | *Pentacta dispar* (Lampert, 1869) |  |  |  |  |  |  | **+** | **+** |  |  | Price, 1982a |
| *Pseudocnus echinatus* (von Marenzeller, 1882) |  |  |  |  |  |  |  | **+** | **+** |  |  | Price, 1982a |
| *Staurothyone rosacea* (Semper, 1869) |  |  |  |  |  |  |  | + | + |  |  | Price, 1982a |
| *Trachasina crucifera* (Semper, 1869) | *Trachythyone crucifera* (Semper, 1869) |  |  |  | + |  |  | + | + |  |  | Price, 1982a |
| *Trachythyone glaberrima* (Semper, 1869) | *Paracucumaria glaberrima* Semper, 1869,  *Trachythyone dollfusi* Cherbonnier, 1954 |  |  |  | + |  |  | + | + |  |  | Price, 1982a |
| **Phyllophoridae** [**Östergren, 1907**](about:blank) |  |  |  |  |  |  |  |  |  |  |  |  |
| *Phyrella tenera* (Ludwig, 1875) | *Semperiella tenera* (Ludwig, 1875) |  |  |  | + |  |  |  |  |  |  | Price, 1982a |
| *Thorsonia fusiformis* Heding, 1940 |  |  |  |  |  |  |  |  |  | + |  | Heding, 1940; Dabbagh et al., 2012b |
| *Thyone* sp |  |  |  |  | + |  |  |  |  |  |  | Price 1981, 1983 |
| *Thyone dura* Koehler & Vaney, 1908 |  |  |  |  |  |  |  | + |  | + |  | Dabbagh et al., 2012b; Claereboudt & Al-Rashdi, 2011 |
| *Thyone quadriperforata* Cherbonnier, 1954 |  |  |  |  |  |  |  | + | + |  |  | Price, 1982a |
| *Stolus buccalis* (Stimpson, 1855) | *Stolus sacellus* Selenka, 1867 |  |  | **+** |  |  |  | + | + | + |  | Heding, 1940; Dabbagh et al., 2012b |
| **Sclerodactylidae Panning, 1949** |  |  |  |  |  |  |  |  |  |  |  |  |
| *Athyone transitoria* (Vaney, 1905) Cherbonnier, 1955 |  |  |  |  |  |  |  | + | + |  | Taxon inquirendum | Price, 1982a |
| *Havelockia transitoria* (Vaney, 1905) |  |  |  |  |  |  |  | + | + |  |  | Price, 1982a |
| *Havelockia festina* (Koehler & Vaney, 1908) | *Thyone festina* Koehler & Vaney, 1908 |  |  | + |  |  |  |  |  | + |  | Heding, 1940; Dabbagh et al., 2012b |
| *Oshimella ehrenbergii* (Selenka, 1868) |  |  | + |  | + |  |  | + | + | + |  | Price,1983; Al-Yamani et al., 2012; Dabbagh et al., 2012b |
| **Holothuriida Miller, Kerr, Paulay, Reich, Wilson, Carvajal & Rouse, 2017** |  |  |  |  |  |  |  |  |  |  |  |  |
| **Holothuriidae Burmeister, 1837** |  |  |  |  |  |  |  |  |  |  |  |  |
| *Actinopyga bannwarthi* Panning, 1944 |  |  |  |  | + |  |  | + | + |  |  | Price, 1982a |
| *Actinopyga crassa* Panning, 1944 |  |  |  |  |  |  |  | + | + |  |  | Price, 1982a |
| *Actinopyga echinites* (Jaeger, 1833) | *Actinipyga plebeja* (Selenka, 1867) |  |  |  | + |  |  |  |  |  | Commercial value-medium | Hasan, 2008 |
| *Actinopyga lecanora* (Jaeger, 1835) |  |  |  |  |  |  |  | + | + |  | Commercial value-medium to high | Price, 1982a |
| *Actinopyga mauritiana* (Quoy & Gaimard, 1834) |  |  |  |  | + |  |  | + |  |  | Commercially value- medium to high | Hasan, 2008; Claereboudt & Al-Rashdi, 2011 |
| *Actinopyga milliaris* (Quoy & Gaimard, 1834) |  |  |  |  | + |  |  | + |  |  | Commercially value-medium | Price, 1982a; Claereboudt & Al-Rashdi, 2011 |
| *Actinopyga serratidens* Pearson, 1903 |  |  |  |  | + |  |  |  |  |  |  | Price, 1982a |
| *Actinopyga* sp. |  |  |  |  |  |  |  | + |  |  |  | Claereboudt & Al-Rashdi, 2011 |
| *Bohadschia costeaui* Cherbonnier, 1954 |  |  |  |  | + |  |  |  |  |  |  | Price, 1982a |
| *Bohadschia marmorata* Jaeger, 1833 |  |  |  |  | + |  |  |  |  |  | Commercial value- medium | Price, 1982a |
| *Bohadschia ocellata* Jaeger, 1833 | *Holothruia ocellata* Jaeger, 1833 |  |  |  | + |  |  |  |  | + |  | Price, 1982a |
| *Bohadschia vitiensis*  (Semper, 1869) | *Bohadschia tenuissima* (Semper, 1868) |  |  |  | + |  |  |  |  |  | Commercial value- medium | Hasan, 2008 |
| *Holothuria aphanes* Lampert, 1885 |  |  |  |  | + |  |  | + | + |  |  | Price, 1982a |
| *Holothuria albiventer* Semper, 1868 |  |  |  |  | + |  |  | + | + |  |  | Price, 1982a |
| *Holothuria arenacava* Samyn, Massin & Muthiga, 2001 |  |  |  |  |  |  |  | + |  |  |  | Claereboudt & Al-Rashdi, 2011 |
| *Holothuria arenicola* Semper, 1868 |  |  | + |  | + |  |  |  | + | + | Commercial value- low | Basson, 1977; Price 1981, 1982a, 1983; Dabbagh & Kamrani, 2011; Al -Yamani et al., 2012 |
| *Holothuria atra* Jaeger, 1833 |  |  |  |  | + |  |  |  |  | + | Commercial value-medium | Price, 1981, 1983; Dabbagh et al., 2012b |
| *Holothuria bacilla* Cherbonnier, 1988 |  |  |  |  |  |  |  |  |  | + |  | Pourvali & Nabavi, 2014; Afkhami et al., 2015 |
| *Holothuria cinerascens* (Brandt, 1835) |  |  |  |  | + |  |  | + | + |  | Commercial value- low | Price, 1982a; Claereboudt & Al-Rashdi, 2011 |
| *Holothuria difficilis* Semper, 1868 |  |  |  |  | + |  |  |  |  |  |  | Price,1982a |
| *Holothuria edulis* Lesson, 1830 |  |  |  |  | + |  |  | + |  |  | Commercial value- low | Price, 1981, 1982a, 1983; Claereboudt & Al-Rashdi, 2011 |
| *Holothuria flavomaculata* Semper, 1868 |  |  |  |  | + |  |  |  |  |  |  | Price, 1982a |
| *Holothuria fuscocinerea* Jaeger, 1833 |  |  |  |  | + |  |  |  |  |  |  | Price,1982a |
| *Holothuria fuscogilva* Semper, 1868 |  |  |  |  | + |  |  |  |  |  | Commercial value-high | Hasan, 2008 |
| *Holothuria fuscoolivacea* Ludwid, 1868 |  |  |  |  | + |  |  |  |  |  |  | Price, 1982a |
| *Holothuria hilla* Lesson, 1830 | *Holothuria monacaria* Lesson, 1830 |  |  |  | + |  |  | + | + | + | Commercial value-low | Price, 1981, 1982a, 1983; Shakouri et al., 2009; Salarzadeh et al., 2013 |
| *Holothuria glandifera* (Cherbonnier, 1955) |  |  |  |  | + |  |  |  |  |  |  | Price, 1982a |
| *Holothuria impatiens* (Forsskål, 1775) |  |  |  |  | + |  |  | + | + | + | Commercial value-low | Price, 1981, 1983; Dabbagh et al., 2012b; Salarzadeh et al., 2013 |
| *Holothuria inhabilis* Selenka, 1867 |  |  |  |  | + |  |  |  |  |  |  | Price, 1982a |
| *Holothuria insignis* Ludwig, 1875 |  |  |  |  | + |  |  |  |  |  |  | Price, 1982a |
| *Holothuria kurti* Ludwig, 1891 |  |  |  |  | + |  |  |  |  |  |  | Price, 1982a |
| *Holothuria leucospilota* (Brandt, 1835) | *Holothuria vagabunda* Selenka, 1867 |  | + | + | + |  |  | + | + | + | Commercial value-low | Price 1981, 1983; Dabbagh & Kamrani 2011; Afkhami et al., 2012; Salarzadeh et al., 2013; Pourvali et al., 2014; Papathanaspoulou and Zogaris, 2015 |
| *Holothuria martensi* Semper, 1868 |  |  |  |  |  |  |  | + | + |  |  | Price, 1982a |
| *Holothuria nobilis* (Selenka, 1867) |  |  |  |  | + |  |  |  | + |  | Commercial value- high | Price, 1982a; Hasan, 2008 |
| *Holothuria notabilis* Ludwig, 1875 |  |  |  |  |  |  |  |  |  | + |  | Afkhami et al., 2014 |
| *Holothuria papillifera* Heding in Mortensen, 1938 |  |  |  |  | + |  |  |  |  |  | Taxon inquirendum | Price, 1982a |
| *Holothuria pardalis* Selenka, 1867 |  |  |  |  | + |  |  |  | + | + |  | Price, 1982a; Dabbagh et al., 2012b |
| *Holothuria parva* Krauss in Lampert, 1885 |  |  |  |  | + |  |  | + | + | + |  | Price, 1982a, 1983; Dabbagh & Kamrani 2011; Ehsanpour et al., 2016, |
| *Holothuria parvicax* Selenka, 1867 Lampert, |  |  |  |  | + |  |  | + | + |  | Commercial value-low | Price, 1982a; Claereboudt & Al-Rashdi, 2011 |
| *Holothuria remollescens* Lampert, 1885 |  |  |  |  | + |  |  |  | + |  |  | Price, 1982a |
| *Holothuria rigida* (Selenka, 1867) |  |  |  |  | + |  |  |  | + |  |  | Price, 1982a |
| *Holothuria scabra* Jaeger, 1833 |  |  |  |  | + |  | + | + |  | + | Commercial value- high | Price, 1982a; Claereboudt & Al-Rashdi, 2011; Dabbah et al., 2012a; Pourvali & Nabali 2014; Yaghmour & Jones, 2018 |
| *Holothuria spinifera* Théel, 1886 |  |  |  |  | + |  |  |  |  | + | Commercial value-Medium | Dabbagh et al., 2012b |
| *Holothuria strigosa* Slenka, 1867 |  |  |  |  | + |  |  |  | + |  |  | Price, 1982a |
| *Holothuria* sp. |  |  |  |  | + |  |  |  |  | + |  | Price, 1981; Pourvali & Nabavi 2014 |
| *Holothuria tortonesei* Cherbonnier, 1979 |  |  |  |  | + |  |  |  | + |  |  | Price, 1982a |
| *Labidodemas semperianum* Selenka, 1867 |  |  |  |  | + |  |  |  |  |  |  | Price 1981, 1983 |
| *Pearsonothuria graeffei* (Semper, 1868) | *Bohadschia drachi* Cherbonnier, 1954, *Bohadschia graeffei* (Semper, 1868) |  |  | + |  |  |  |  | + |  | Commercial value-low | Price, 1982a |
| **Molpadida Haeckel, 1896** |  |  |  |  |  |  |  |  |  |  |  |  |
| **Caudinidae Heding, 1931** |  |  |  |  |  |  |  |  |  |  |  |  |
| *Acaudina leucoprocta* (H.L. Clark, 1938) | *Aphelodactyla irania* Heding, 1940 |  |  |  |  |  |  |  |  | + |  | Dabbagh et al., 2012b |
| **Synallactida Miller, Kerr, Paulay, Reich, Wilson, Carvajal & Rouse, 2017** |  |  |  |  |  |  |  |  |  |  |  |  |
| **Stichopodidae Haeckel, 1896** |  |  |  |  |  |  |  |  |  |  |  |  |
| *Stichopus chloronotus* Brandt, 1835 |  |  |  | + |  |  |  |  | + |  |  | Price, 1982a |
| *Stichopus hermanni* Semper, 1868 | *Stichopus variegatus* Semper, 1868 |  | + |  | + |  |  | + | + | + | Commercial value-high | Price,1982a, 1983; Afkhami et al., 2012, 2015, Papathanaspoulou and Zogaris, 2015 |
| *Stichopus horrens* (Selenka, 1867) |  |  | + |  |  |  |  |  |  | + | Commercial value-medium | Papathanaspoulou and Zogaris, 2015; Noura et al., 2019 |
| *Stichopus* cf *monotuberculatus* (Quoy and Gaimard, 1834) |  |  |  | + |  |  |  | + | + | + | Commercial value-high | Price, 1982a; Salarzadeh et al., 2013; Ehsanpour et al., 2012; Salarzadeh et al., 2013, Noura et al., 2019 |
| **Apodida Brandt, 1835** |  |  |  |  |  |  |  |  |  |  |  |  |
| **Synaptidae Burmeister, 1837** |  |  |  |  |  |  |  |  |  |  |  |  |
| *Euapta godeffroyi* (Semper, 1868) |  |  |  |  | + |  |  | + | + |  |  | Price, 1982a; Claereboudt & Al-Rashdi, 2011 |
| *Leptosynapta chela* Mortensen, 1926 |  |  |  |  | + |  |  |  |  |  |  | Price, 1981 |
| *Leptosynapta steinitzi* Cherbonnier, 1967 |  |  |  |  | + |  |  |  | + |  |  | Price, 1982a |
| *Ophiodesoma grisea* (Semper, 1867) |  |  |  |  | + |  |  | + | + |  |  | Price,1982a |
| *Ophiodesoma kamaranensis* Clark in Clark & Rowe, 1971 |  |  |  |  | + |  |  |  | + |  |  | Price, 1982a |
| *Patinapta crosslandii* Heding, 1929 |  |  |  |  | + |  |  |  | + |  |  | Price, 1982a |
| *Polyplectana keferstienii* (Selenka, 1867) |  |  |  |  | + |  |  |  | + |  |  | Price, 1982a |
| *Protankyra magnihamula* Heding, 1928 |  |  |  |  |  |  |  |  |  | + |  | Heding, 1940; Dabbagh et al., 2012b |
| *Protankyra pseudodigitata* (Semper, 1867) |  |  |  |  |  |  |  |  |  | + |  | Heding, 1940; Price, 1981, 1983; Dabbagh et al., 2012b; Peygham et al., 2018 |
| *Synapta maculata* (Chamisso & Eysenhardt, 1821) |  |  |  |  | + |  |  | + | + |  |  | Price, 1982a |
| *Synaptula reciprocans* (Forsskål, 1775) |  |  |  |  | + |  |  | + | + |  |  | Price, 1982a |
| *Synaptula recta* Semper, 1869 |  |  |  |  | + |  |  |  | + |  |  | Price, 1982a |
