# Supplementary material for: Sea cucumbers of the Arabian Peninsula and Iran – A review of historical and current research trends
Source: Saudi J Biol Sci. 2021 Oct 6;28(11):6116–26. doi: 10.1016/j.sjbs.2021.10.001 (PMC8568818; doi:10.1016/j.sjbs.2021.10.001)
Supplement: Supplementary data 2 [file mmc2.docx]

Supplementary Table S2. Recent bioprospecting studies in Sea cucumbers of Iran.

| Property | Species | Description |  |  | Reference |
| --- | --- | --- | --- | --- | --- |
| Antifungal | *H. leucospilota* | Anti-fungal properties with prospects to develop anti-fungal drugs. |  |  | Mokhlesi et al., 2012 |
|  | *H. leucospilota* | Anti-fungal activity of Methanol extracts of the body wall against *Aspergillus niger* and exhibits high cytoxicity.  Body wall extracts showed strong anti-fungal activity against *Aspergillus* *niger*, at a concentration of 18 µg/ml |  |  | Mohamaddizadeh et al., 2013  Sarhadizadeh et al., 2014 |
|  | *S. hermannii* | Steroidal and Glycosidal-Steroidal Saponins exhibits immense activity against *Aspergillus fumigatus* and *Candida albicans.* |  |  | Zhao et al., 2018 and Zahra et al., 2017 |
| Antibacterial | *H. leucospilota* | Compared to aqueous, Methanol extracts were very active against gram negative bacteria. |  |  | Nazemi et al., 2016 |
|  | *H. parva* | Bactericidal properties against human pathogens such as *Escherichia coli, Pseudomonas aeruginosa, E. faecalis*. |  |  | Ebrahimi et al., 2018 |
|  | *H. leucospilota* | β amberine extracted from the gonad exhibited antibacterial properties against *Bacillus aureus*, *B. subtilis*, *Salmonella typhi* and *Staphylococcus aureus* with a potential to develop a novel antibiotic. |  |  | Nazemi et al., 2019 |
|  | *S. hermannii* | Steroidal and glycosides-steroidal saponins inactive against gram negative bacterium *Pseudomonas aeruginosa*, active against gram positive *Staphylococcus aureus*. |  |  | Zahra et al., 2017 |
|  | *H. leucospilota* | Tested against *Pseudomonas*, a major contaminant in frozen Salmon fillets due to its H_2_S producing capabilities. Extracts at 2 % concentration showed a very high antibacterial activity with potential to act as a preservative for frozen fish products. |  |  | Hannaneh et al., 2016 |
|  | *H. leucospilota and Niphates furcata* (Sponges) | Comparative antibacterial activity of methanol extracts indicated that, extracts of the sponge *N. furcata* was active against gram negative and positive bacteria than *H. leucospilota*. |  |  | Nazemi et al., 2017 |
|  | *H.parva* | Body wall extracts possess very intense antibacterial activity against human pathogens: *Escherichia coli*, *Vibrio parahaemolyticus* and *S. aureus C*ytotoxic and hemolytic properties were also observed. |  |  | Shadi and Oujifard, 2019 |
| Antiviral | *H. leucospilota*  *H. leucospilota* | Holostane -type triterpenoids extracted showed moderate cytotoxicity  Methanol extracts showed no activity against HIV due to its cytotoxic effects. |  |  | Shushizadeh et al..2019  Bahroodi et al., 2016 |
|  | *H. leuospilota* | Diethyl extracts of digestive glands and body wall showed strong antiviral (HIV) and cytotoxic activity. |  |  | Bahroodi et al., 2018 |
| Anticancer | *H. leuospilota* | Sea cucumbers contain Chondroitin sulphate which is effective in the treatment of bone defects, further alcohol extracts differentiated stem cells extracted from rat bone marrow into osteogenic and adipogenic at a concentration of 25 µg/l. |  |  | Baharara et al., 2014 |
|  | *H. leucospilota* | Methnol and diethyl ether extracts shows high cytotoxic activity against human embryonic kidney cell line (HEK) and arrested growth in oral carcinoma (KB) and Melanoma cell lines. |  |  | Bahroudi et al., 2016 |
|  | *H. leucospilota* | Induced apoptosis in melanoma cell lines. |  |  | Nikdel et al., 2015 |
|  |  |  |  |  |  |
|  |  | Extracts effective in inhibiting the growth of cervical cancer cells. |  |  | Baharara et al., 2016a |
|  |  | Saponin extracts combination with Dacarbazine has effective anti-cancer property against B16F10 melanoma cell line. |  |  | Baharara et al., 2016b |
|  | *H. arenicola* | Aqeous extracts were active against CT26 carcinoma cells. |  |  | Baharara et al., 2016c |
|  | *H. leucospilota* | Bacteria such as *Streptomyces* sp. isolated from the gut also possess antioxidant and cytotoxic effects. |  |  | Gozari et al., 2018 |
|  | *H. leucospilota, H. parva and H. scabra* | Strong anti-cancer property with potential for drug development. |  |  | Seydi et al., 2015, Soltani and and Baharara, 2014, Soltani et al., 2015 Mashjoor and Yousefzahdi, 2017, Mashjoor et al., 2019 |
|  | *H. leucopilota* | High antioxidant and anti-tumor properties against breast cancer cell line by suppressing cell viability, VEGF-D and TGFβ expression. |  |  | Soltani et al., 2015b |
|  | *S. hermanni* | Pure fractions of Saponins can be extracted through thin layer chromatography. |  |  | Zahra et al., 2017 |
| Biochemical | *H. leucospilota and H. scabra* | High levels of ω-3 fatty acids identified |  |  | Yahavi et al., 2012 |
|  | *H. scabra* | Contains high Heneiocosanoic acid with pharmaceutical and cosmetic value. |  |  | Jadavi et al., 2016 |
|  | *Stichopus horrens* | Type 1 collagen content with high quality collagen compared to fishes and mammals. |  |  | Attaran et al., 2016 |
|  | *H. leucospilota* and *S. hermmani* | High protein (30-33%), Amino acid (Aspartic Acid) and low-fat content is available in dried sea cucumbers used for human consumption. |  |  | Moradi et al 2019 |

**References**

1. Adibpour, N., F. Nasr, F. Nematpour, A. Shakouri, and A. Ameri. Antibacterial and antifungal activity of *Holothuria leucospilota* isolated from Arabian Gulf and Oman Sea. Jundishapur. *J. Microbiol.,* 1: e8708 (2014). https: //DOI:10.5812/jjm.8708.
2. Ahmed, S., and E. Khanonmnaz. Comparison of antioxidant capacity of dry and wet sea cucumber *Holothuria leucospilota* tissue*. J. Anim Env*., 10: 471 – 476 (2018).
3. Attaran, F. G., Taheri, A and N. Barzkar. Sea cucumber (*Stichopus horrens*) body wall collagen of Chabahar bay and its gelatin properties. *Ir. J. Food.Sc. Tech.,* 52: 79-89 (2016).
4. Baharara, J., Amini, E., Namvar, F. and M. Soltani. The effect of Arabian Gulf sea cucumber alcoholic extract on osteogenic and adipodgenic differentiation of rat mesenchymal stem cells. *J. Cell and Tissue*, 5:273-280 (2014).
5. Bahrara, J., Amini, E. and V. Vazifedan. Concomitant use of sea cucumber organic extract and Radiotherapy on proliferation and apoptosis of cervical (HeLa)cell line. *Zahedan J. Res. Med. Sci.,* 18: e6422 (2016a). doi: 10.17795/zjrms-6442.
6. Bahrara, J., Amini, E., Nikdel, N. and F.S. Abdollahi. The cytotoxicity of Dacarbazine potentiated by sea cucumber saponin in resistant B16F10 melanoma cells though Apoptosis induction. *Avi, J. Med. Biotech*., 8: 112-119 (2016b).
7. Baharara, J. Amini, E., Afzali, M., Nikdel, N., Mostafapour, A., And M.A. Kerachian. Apoptosis inducing activity of *Holothuria arenicola* in CT26 colon carcinoma cells *in vitro* and *in vivo. Ir. J. Basic. Med. Sci*., 19: 358-365 (2016c).
8. Bahroudi, S. Nemottolahi, M.A., Sadeghi, M. R. A., Nazemi, M. and B Behrooz. Anti-viral effect of methanolic extract of Sea cucumber on HIV-1 virus. *J. Gorgan Univ. Med. Sci.,* 17: 125-131 (2016).
9. Bahroudi S, Nematollahi M.A, Aghasadeghi M.R, Nazemi M. In vitro Evaluation of the Antiviral Activity and Cytotoxicity Effect of *Holothuria leucospilota* Sea Cucumber Extracts from the Arabian Gulf. *Inf. Epidem. Microbiol*., 4: 153-157 (2018).

and

1. Dobretsov, S., Al-Mammari, I.M., Soussi, B. Bioactive compounds from Omani sea cucumbers. *Agri. Mar. Sci*., 14, 49-53, (2009).
2. Ebrahimi, H., Vazirizadeh., A., Nabipour, I. Najafi, A., Tajabaksh, S. and B.M. Nafisi. In vitro study of antibacterial activities of ethanol, methanol and acetone extracts from sea cucumber *Holothuria parva. Ir. J. Fish. Sci*., 17: 543- 551 (2018). DOI:10.22092/IJFS.2018.116472
3. Ghadiri, M., S. Kazemi, B. Heidari, and M. Rassa. Bioactivity of aqueous and organic extracts of sea cucumber *Holothuria leucospilota* (Brandt, 1835) on pathogenic *Candida* and *Streptococci.* *Int. Aquat. Res*., 10: 31-43 (2018). <https://doi.org/10.1007/s40071-017-0186-x>
4. Gozari, M., Bahador, N., Jassbi, A.R. Mortazavi, M.S. and E. Eftekhar. Antioxidant and cytotoxic activities of metabolites produced by a new marine *Streptomycetes* sp. isolated from the sea cucumber *Holothuria leucospilota*. *Ir. J. Fish.Sci.,* 17: 413-426 (2018). DOI: 10.22092/IJFS.2018.116076
5. Hannaneh, R., Vali, H.S., Ali, M.M.A. and M.A Reza. Evaluation of the antimicrobial activity of sea cucumber (*Holothuria leucospilota*) extract on the shelf -life of rainbow trout (*Onchorhynchus mykiss*) fillets during refrigerated storage. *J. Fish. (Ir. J. Nat. Res.),* 69: 29-37 (2016).
6. Jadavi, N., Vaziri, S.A. Nabipour, I., Jafari Nasr, M.R. and G.H. Mohebbi. Fat characteristics and fatty acid profiles of sea cucumber *Holothuria scabra* obtained from the coasts of Bushehr province-Iran. *Ir. South Med. J.,* 18: 992-1006 (2015). DOI: 107.508/ismj.1394.05.007
7. Jamali, S., E. Hossein, T. L. Teymouri, Z. Sirous, S. Keypour, S. Sardari, R. Ali and A. Parisa. Antibacterial effect of the Arabian Gulf sea cucumber *Holothuria sp* extracts on three strain of Escherichia coli. *Modares J. Med. Sci*., (Pathobiology), 12: 37 – 49 (2009).
8. Mashjoor, S. and M. Yousefzadi. Cytotoxic effects of the three Arabian Gulf species of Holothurians. *Ir. J. Vet. Res.,* 20:19-26 (2019).
9. Mashjoor, S., Yousefzadi, M. and F. Pishevarazad. Assessment of anticancer potential of selected *Holothuria species*. *Ind. J. Trad. Know.,* 18:272-280 (2019). http://nopr.niscair.res.in/handle/123456789/47085
10. Mohammadizadeh, F., Eshanpour, M, Afkhami, M., Mokhlesi, A, Khazali, A. and S. Montazeri. Antibacterial, antifungal and cytotoxic effects of a sea cucumber *Holothuria leucospilota*, from the north coast of the Arabian Gulf. *J. Mar. Biol Assoc. U.K*., 93: 1401-1405 (2013). doi:10.1017/S0025315412001889
11. Moradi, Y., Nazmi, M., Ghaeni, M., & Lakzaei, F. and S. Sharokhi. Proximate composition and amino acid contents of two sea cucumbers, *Holothuria leucospilota* and *Stichopus herrmanni.* *J. Fish* (Ir. J. Nat. Res.), 72: 97-10 (2019).
12. Nazemi, M., Moraidy, M., Gazari, S., Legazee, F. and M.Karimpoor. Investigations of antibacterial activity of Methanol and aqueous extracts of the body wall of sea cucumber *Holothuria Leucospilota* on some human pathogenic bacteria. *Avi. J. Clin. Med*., 23: 75-82 (2016).
13. Nazemi, M., Tamadoni, J.S., Salari, Z. and M. Gozari. Comparison of antibacterial activity of methanol extract of sea cucumber (*Holothuria leucospilota*) and sponge *Niphates furcata* from Hengam island, Arabian Gulf. *J. Mar. Biol.,* 8: 65-72 (2017).
14. Nazemi, M., moradi, Y., Ghaffari, H. and J. K. Kevian. The antibacterial activity of β amberine extracted from the gonad of sea cucumber *Holothuria leucospilota* inhabiting Hengam island, Arabian Gulf. *J. fish* (*Ir. J. Nat. Res.*)., 72: 195-204 (2019).
15. Nikdel, N., Baharara, J. Nejad, S.K. and E. Amini. The cytotoxic effects of sea cucumber body wall extract, species of *Holothuria arenicola* melanoma cells. *J. Shahrekord Univ. Med. Sci.,* 17: 33-43 (2015).
16. Mokhlesi, A. Saeidnia, S, Gohari, A.R., Shahverdi, A.R., Nasrolahi, A., Farahni, F, Khoshnood, R. and N. Es’hagi. Biological activities of the sea cucumber *Holothuria leucospilota*. *Asian J. Anim. Vet. Sci.,* 7: 243-249 (2012). DOI: 103923/ajava.2012.243.249
17. Sarhadizadeh, N., Afkhami, M. and M. Eshanpour. Evaluation of bioactivity in sea cucumber, Stichopus hermanni from Arabian Gulf. *Eur. J. Exp. Biol*., 4: 254-258. (2014).
18. Shadi, A. and A. Oujifard. Antibacterial, cytotoxic and hemolytic activity of *Holothuria parva* sea cucumber from north Arabian Gulf. *Int. J. Environ. Sci. Technol.,* 16, 5937–5944 (2019). <https://doi.org/10.1007/s13762-018-1956-8>
19. Shushizadeh, M.A., Pour, P.M., Mahdeih, M. and Yagdaneh, A. Phytochemical analysis of *Holothuria leucospilota*, a sea cucumber from Arabian Gulf. *Res. Pharm. Sci*., 14: 442-440 (2019). doi: [10.4103/1735-5362.268204](https://dx.doi.org/10.4103%2F1735-5362.268204)
20. Shakouri, A., M. R. Shoushizadeh, and F. Nematpour. Antimicrobial activity of sea cucumber (*Stichopus variegatus*) body wall extract in Chabahar Bay, Oman Sea. *Jundishapur J. Nat. Pharm. Prod*. ,12: e32422 (2017). https://doi: 10.5812/jjnpp.32422
21. Seydi, E., Motallebi, A., Dastabz, M., Dehghan, S., Salimi, A., Nazemi, M. and J. Pourahamad.

Selective Toxicity of Arabian Gulf Sea Cucumber (*Holothuria parva*) and Sponge (*Haliclona oculata*) Methanolic Extracts on Liver Mitochondria Isolated from an Animal Model of Hepatocellular Carcinoma *Hepat. Mon*., 15: e33073 (2015). doi: 1.5812/hepatmon.33073.

1. Soltani M and J. Baharara. Antioxidant and antiprolifereative capacity of dichloromethane extract of *Holothuria leucospilota* sea cucumber. *Int J Cell. Mol. Biotech.,* 2014: 1-9 (2014). doi:10.5899/2014/ijcmb-00013
2. Soltani, M., Parivar, K. Baharara, J., Kerachian, M.A. and J. Asili. Putative mechanism for apoptosis-induction properties of crude saponin isolated from sea cucumber (*Holothuria leucospilota*) as an antioxidant. *Ir. J. Basic Med. Sci.,* 18:180-187 (2015a).
3. Soltani, M., Parivar, K. Baharara, J., Kerachian, M.A. and J. Asili. Transcriptional analysis of VEGF-D and TGFβ in MCF7 cells exposed to Saponin isolated from *Holothuria leucospilota* (sea cucumber). *Rep. Biochem. Mol.Biol*.,4: 1-7 (2015b).
4. Yahavi, M., Afkhami, M, Javadi, A., Ehsanpour, M., Khazaali, A., Khishnood, R and A. Mokhlesi. Fatty acid composition in two sea cucumber species, *Holothuria scabra* and *Holothuria leucospilota* from Qeshm island (Arabian Gulf). *Afr. J. Biotech.*, 11: 2862-2668. DOI: 10.5897/AJB11.3529
5. Zahra,S., Sourinejad, I., Nazemi, M and M. Yousefzadi. Qualitative survey of the extracted saponin from the Arabian Gulf sea cucumber *Stichopus hermanni*. *J. Aquat. Physiol. Biotech.,* 5: 21-36 (2017).
6. Zhao, Y. C., Xue, H.C., Zhang., T.T. and Y.M. Wang. Saponins from sea cucumber and their biological activities. *J. Agr. Food Chem.*, 66: 7222-7237 (2018). DOI: 10.1021/acs.jafc.8b01770
